# Mr. Graham's Case of Enterodynia

**Published:** 1808-06

**Authors:** P. Graham

**Affiliations:** Assistant Surgeon, 22d Regt. Great Russel Street, Bloomsbury Square


*May* 14, 1808.

---

### *To the Editors of the Medical and Physical Journal.*

GENTLEMEN,

THE following singular Case occurred while I was attending my medical studies, &c. in Edinburgh, in 1799. I have neither read nor seen of any case of the kind in this country, although I have been told such a one has appeared on the Continent. Should you deem it worthy a place in your truly valuable Journal, its insertion will oblige

Yours, &c.

P. GRAHAM,
Assistant Surgeon, 22d Regt.

*Great Russel Street, Bloomsbury Square,*

*May* 14, 1808.

---

Mr. FRANCIS BELL, æt. 20. February 1, 1799.—Over all his extremities there is an eruption of red pimples, like to that of small-pox, but more livid and broader in their bases. These, he says, give a kind of prickly sensation similar to what is felt after being rubbed with nettles.

No cause can be assigned for this eruption except exposure to cold, and afterwards being much heated; is not certain but he got a drink of small beer, and on the succeeding day the spots appeared, which, from the uneasiness they occasioned, he exposed to a free current of cold air, and finding some relief, he washed his legs for some time with cold water and snow. Took ʒij. or iij. of cream of tartar, and a little while after, felt some pain in his intestines like cholic, which he attributed to the cream of tartar.

2d. Pain

2d. Pain of bowels more severe; the spots increasing in number and size; rested pretty well during the night. Took a draught of æther and laudanum about ten o'clock at night for the pain in his belly, but found no benefit from it. To day and yesterday, a kind of uneasy sensation was felt over his whole body, which he attributes to the eruption. Belly bound; urine high coloured.

3d. Pain of the belly increasing; belly still bound, and urine high coloured. Took his breakfast pretty well, but immediately after was seized with sickness at stomach, and became quite uneasy and restless during the remaining part of the day; was seized with vomiting about twelve at noon, and again about nine at night. The matter ejected was tinged with green, and of a bad smell.

4th. Rested badly during the night; pain of belly still more severe; belly still costive; is affected with hiccup, alternating with constant nausea and vomiting of bilious matter, and every thing taken into the stomach is immediately ejected; urine high coloured. Dr. Monro being called in, ordered him a common enema and an infusion of senna if the injection did not operate, both of which he got without effect; was ordered likewise to drink plentifully of beaf-tea and water-gruel.

5th. Symptoms same as yesterday. The spots still growing larger and more plentiful, and on the back part of the right humerus and right buttock they are run into one another, and from being of a deep red, they now put on the appearance of a dull purple; those on the legs, thighs, and left arm are distinct from one another; complains of great difficulty in making water. As Dr. Monro did not visit him to day, Dr. Hope was called in about nine at night, and ordered his belly to be fomented till he should make water freely; was likewise ordered an infusion of senna, a tea-cup full at a time, to be taken occasionally till it operated, and if it did not operate, to get an injection the same night about ten o'clock. Pulse about 90, and small; skin rather cold.

6th. Neither the senna nor injection operated; was visited by Dr. Monro, who ordered the following injection and pills.

℞. Fol. sennæ ʒij. infund. in aq. bullient. ℔i. et adde. Sal. Glauber. ℥ß. ol. olivar. ℥i. M. ft. Enema statim injicend. et hora 10ma. rep. nisi prius.

℞. Calomel gr. xii. Micæ panis ʒß. Syr. simpl. Q. S. M. et divid. in pil. vi. quarum unam sumat alterâ quâque horâ.

Vomiting

Vomiting of bilious matter, nausea, and hiccup, still continue; urine high coloured; sometime after Dr. Monro's departure, was visited by Dr. Hope, who approved of Dr. Monro's prescription. Bad sleep during the night. Pulse and skin as on the preceding day.

7th. A second injection was given this morning, which operated very well. Pain of stomach, intestines, nausea, vomiting, and hiccup nearly gone; urine made with ease after the fomentation, still high coloured. A gentle diarrhœa came on to day about noon; some small spots now appearing on his *nares* and eyebrows. To have ℔ij of wine in the twenty-four hours. Bad sleep in the night. Omitt. pil. Pulse between 80 and 90, and more feeble; skin and feet cold.

8th. Slept pretty well during the night; diarrhœa nearly gone. The effusion increasing on his face, about the *nares*, eyebrows, and cheeks, and growing more livid.—Those on the other parts of his body nearly the same, a little more livid in appearance.

9th. Pulse fuller and more regular, above 110; skin warm; face and hands considerably swelled, and of a somewhat redder colour between the purple spots than natural; some sleep in the night; on the whole, seems to be a little fresher. The effusion and spots do not decrease, and there is the appearance of two small vesicles on the effused parts of the right humerus, and slight effusion on the tunica albuginea of the left eye; was seized with slight delirium on the afternoon of this day. Nausea and vomiting nearly gone; belly gently loose; stools very thin and fœtid; urine still high coloured, and deposits a slight reddish tinge upon the sides of the pot immediately after being voided, and there is an appearance like to oily particles floating above it; was ordered by Dr. Hope ℥xii. of wine in the day, and a mixture with sulph. acid; the latter of which by mistake was not given.

10th. Pulse pretty regular, of good strength, towards 120; slight delirium through the night; some vomiting of bile; was ordered by Dr. Hope a calomel pill of gr. iv. and to be repeated in the evening, if the former did not operate. Pill operated, and produced three pretty copious thin fœtid stools.

Ten at night. More delirium, and very restless; constant tossing in bed; complains of great pain at his heart, and the sensation of a lump in his throat, as he says. Blisters over the effused parts are increasing, (and seem to be going rapidly to gangrene;) more effusion on his face; constant pervigilium; lium; strength seems visibly much impaired; great difficulty in swallowing any kind of liquid from the lump in his throat, as he says. Became a great deal calmer after eight in the morning, and seemed pretty distinct; was visited by Dr. Hope about ten, who thinks him in very great danger; continued calm till about twelve, when difficulty of breathing came on, with greater difficulty of swallowing, which, he says, proceeds from the lump in his throat, increasing difficult expectoration of very viscid mucus. Pain in his throat became so severe sometimes, as to require three people to hold him in bed. The effused parts, over the whole back of the humerus, are now covered with blisters, (and complete gangrene seems to have taken place); was ordered by Dr. Hope, ℥i. of bark to be infused into a bottle of wine, a table spoonful of which to be taken frequently.

From twelve till half past one continued quite restless; at times feverish, from the pain of his throat, although pretty distinct when he died.

Was not permitted to inspect the body after death.

